# From Biobank to “Bio-Think-Tank”: Functional Evolution of UK Biobank in Public Health Policy Making

**DOI:** 10.3389/phrs.2026.1609119

**Published:** 2026-03-18

**Authors:** Jun Yin, Antoine Flahault

**Affiliations:** 1 International Law Department, Guangdong University of Foreign Studies, Guangzhou, China; 2 The Institute of Global Health, Faculty of Medicine, Universite de Geneve, Geneva, Switzerland

**Keywords:** bio-think-tank, evidence-based policymaking, functionalist theory, global health governance, UK biobank

## Abstract

**Objectives:**

This study aims to explore the evolving role of the UK Biobank in public health policymaking, particularly its shift from a traditional biobank to a proactive “bio-think tank” and highlight the drivers and necessary elements of this evolution, with a focus on improving its functional impact on policymaking.

**Methods:**

Searches were performed in PubMed, Scopus and Google Scholar databases (end of search: August 20, 2025). We followed guidelines for the PRISMA. We identified 683 potentially relevant titles in our search and selected 51 studies in our review, reflecting “bio-think-tank” issues between January 2004 and August 2025.

**Results:**

Using a functionalist framework, the study identifies key factors driving the UK Biobank’s evolution: limited real-time decision support, rising stakeholder expectations, and the UK’s desire to lead in biobank science. It defines the “bio-think tank” concept and contrasts it with traditional biobanks.

**Conclusion:**

The UK Biobank’s evolution should focus on improving data quality, public trust, accessibility for policymakers, and cross-sector collaboration to strengthen its role as a national research hub and global model for integrating biobank resources with health governance.

## Introduction

The UK Biobank is a large-scale prospective cohort infrastructure established between 2006 and 2010, funded by the UK government and international agencies. The primary objective of the study is to integrate genomic data, multimodal imaging, biomarkers, lifestyle information, and long-term health tracking from approximately 500,000 UK-based volunteers aged 40–69 years. This integration aims to elucidate the interplay between genetic and environmental factors in the development of diseases such as cancer, cardiovascular disease, and neurodegenerative disorders.

The UK Biobank represents not only a “gold mine” for biomedical research but also a critical driver in the transition of public health policy from experience-based to evidence-based decision-making. Through legally compliant data governance, the integration of interdisciplinary resources, and a commitment to global collaboration, it has become an indispensable infrastructure for addressing population aging, chronic disease, and emerging health threats. Looking forward, the completion of whole-genome sequencing and the integration of real-time data from wearable technologies are expected to further advance the paradigm of “precision governance” in public health, reinforcing the UK Biobank’s role as a cornerstone for evidence-informed policymaking and global health innovation [1]. UK Biobank constitutes a powerful engine for epidemiological research and translational science that underpins evidence-informed public health policy making for decades to come [2].

Existing scholarship has extensively examined the UK Biobank in relation to data governance [3–5], consent mechanisms, [6–8], population coverage limitations [9, 10], equity, [11, 12], public interest protection [13, 14], and global cooperation etc. [15, 16] By contrast, far less attention has been devoted to a critical yet underexplored question: how the UK Biobank might transcend its traditional role as a biobank to evolve into a bio-think tank—thereby enabling the design of public health policies that are more scientifically robust, socially responsive, and precisely targeted.

A Bio-think-tank can be defined as an advanced biomedical infrastructure that transcends the traditional role of biobanks as passive repositories of biological samples and health data. Unlike conventional biobanks, which primarily support hypothesis-driven scientific research, a bio-think-tank actively integrates multidimensional data analytics, policy translation mechanisms, and governance frameworks to function as a strategic knowledge hub for public health decision-making.

This study employs a functionalist framework to conceptualize the bio-think-tank through three analytical dimensions: functional cognition, functional hypothesis, and functional performance. It contrasts the fundamental differences between traditional biobanks and bio-think-tanks, elucidating why the UK Biobank must undergo a transition from a conventional biobank to a bio-think-tank in order to achieve functional evolution. The analysis further examines the pathways through which this pivotal transformation can be effectively realized. The findings aim to support policymakers, researchers, and stakeholders in reassessing the policy-relevant functions of the UK Biobank, while also offering international health policymakers a conceptual framework to understand the functional evolution and future trajectory of national biobanks.

### Theoretical Framework: A Three-Dimensional Interpretation of the Functionalist Theory

Based on insights from the functionalist theory, “function” is understood as the utility produced by the item to the judgment and behavior of the relevant actor [17, 18]. From the function of public policy perspective, actors are defined by three key dimensions: functional cognition, functional assumption, and functional performance [19–21]. Functional cognition refers to the cognition of the agent on the nature and characteristics of the object, while functional assumption can be described as the expectation of the agent on the utility of the object. Functional performance is the agent’s assessment of the item. In addition, research in public health and health law demonstrates that policymaking is inherently value-laden, with normative commitments shaping both the production of research and the formulation of policy. Values thus play a pivotal role in guiding decision-making processes and influencing policy outcomes [22, 23]. Judicial and administrative discretion and enforcement factors of legal policies need to be incorporated into the theoretical framework [24], a range of factors such as inter-state competition and national security may hinder or promote the behavior of state actors [25–27].

For this paper, the functional cognition, functional assumption and functional performance of actors on UK Biobank constitute the core concerns, and relevant obstacles and promoting factors should also be investigated to form a theoretical analysis framework. From a functionalist perspective, the UK Biobank’s role is understood through functional cognition, assumption, and performance, influenced by structural obstacles and enabling conditions.

Functional cognition in this context refers to how various stakeholders—including researchers, policymakers, the general public and private companies—perceive the nature and characteristics of the UK Biobank. Studies highlight that the UK Biobank is widely recognized not merely as a genomic database, but as a comprehensive resource integrating multimodal imaging, biomarkers, lifestyle factors, and health outcomes. This breadth of data collection has been described as enabling “population-level precision medicine”, allowing for the dissection of complex gene–environment interactions [28]. Cognitively, scholars see the UK Biobank as an “infrastructural pivot” for linking biological research with actionable health policy [29].

Functional assumption—the expectations actors place on the UK Biobank—has been the subject of both optimism and caution in the literature. On the optimistic side, the UK Biobank is assumed to provide evidence for targeted disease prevention, health inequality monitoring, and early policy intervention [30]. Policymakers expect that its data will underpin AI-driven models for predictive healthcare. However, some scholars warn that such assumptions may overestimate the translational speed from research to policy, especially in the absence of adequate frameworks for bias detection, public engagement, and equitable data governance [31].

Functional performance reflects how well the UK Biobank delivers on its promises. Empirical assessments have shown its exceptional contribution to research productivity, with thousands of publications citing UK Biobank data [32]. Yet, performance is uneven across domains: while the UK Biobank excels in enabling genomic discovery, its integration into policymaking remains partial, hindered by the time lag between scientific discovery and legislative uptake. Additionally, challenges such as selection bias—due to overrepresentation of white, health-literate individuals—limit its representativeness for nationwide policy applications [9].

**FIGURE 1:**
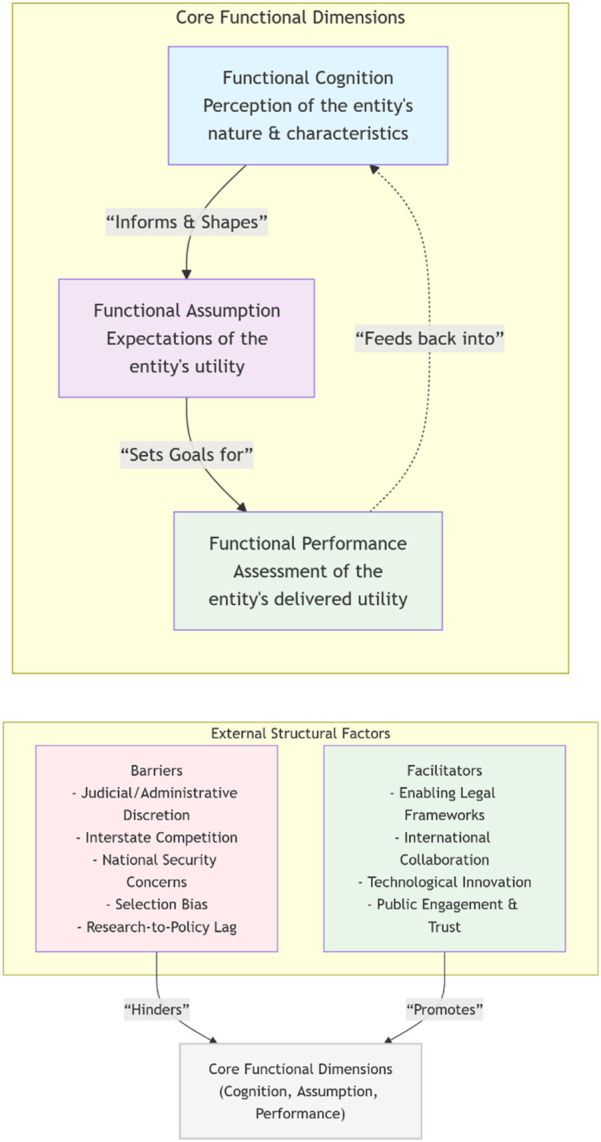
Theoretical framework: A three-Dimensional functionalist analysis (Switzerland, 2025).

In addition to these three functional dimensions, structural factors critically shape the evolution of the UK Biobank. Judicial and administrative discretion directly affects the terms of data-sharing and the viability of cross-border collaborations. Meanwhile, inter-state competition creates both external pressures and incentives for institutional innovation. National security concerns over sensitive genetic information further complicate regulatory approaches, underscoring the intersection of science, law, and geopolitics in the governance of biobanks [33]. The relationship of core functional dimensions and external structural factors have been illustrated in Figure 1.

In sum, scholarly discourse increasingly depicts the UK Biobank as a multifunctional entity whose cognitive, assumptive, and performance-oriented roles are inseparable from broader legal, political, and geopolitical dynamics. A functionalist perspective highlights that its utility is not fixed but continuously co-constructed through the expectations, evaluations, and constraints of diverse stakeholders. Recognizing these layered functions offers a sharper analytical lens for understanding the UK Biobank’s potential transformation from a traditional repository of biological samples into a bio-think-tank capable of shaping health policy at both national and global levels.

## Methods

### Search Strategy and Screening Process

**FIGURE 2:**
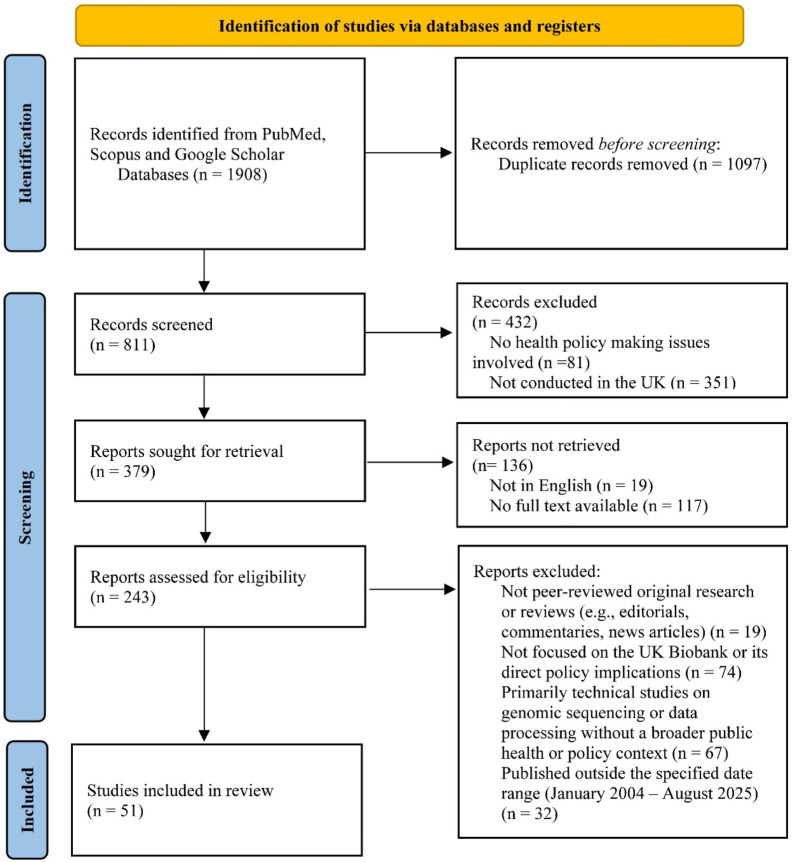
Preferred reporting Items for Systematic Reviews and Meta-Analyses flow diagram (Switzerland, 2025).

The present systematic research was conducted following the Preferred Reporting Items for Systematic Reviews and Meta-Analyses (PRISMA) guidelines (Figure 2) [34]. A systematic literature search was performed in PubMed, Scopus and Google Scholar databases (end of search: August 20, 2025) using the search terms and boolean operators: (UK Biobank) AND public health policy AND (biobank OR bioethics OR policy making OR health law OR health regulation OR global health governance). The respective search was refined to the title-abstract-key word sections in PubMed and Scopus (vs) all article sections in Google Scholar. The Google Scholar articles were sorted by best matching and the first 1000 hits were screened. The reference lists of all eligible articles were systematically searched following a snowball procedure. The search was restricted to articles written in English.

A two-phase screening process took place. Initially, the article abstracts were read independently by two reviewers to decide which papers would be included. Article abstracts (a) the functions of UK Biobank in the formulation of public health policies, and/or (b) The loopholes and deficiencies of UK biobank when used for formulating public health policies and/or (c) The role of biobanks in responding to transformations in global health governance (2nd phase) screening process. We systematically excluded articles that were: (1) not peer-reviewed original research or reviews (e.g., editorials, commentaries, news articles); (2) not focused on the UK Biobank or its direct policy implications; (3) primarily technical studies on genomic sequencing or data processing without a broader public health or policy context; or (4) published outside the specified date range (January 2004 – August 2025). This systematic approach aimed to ensure the relevance and quality of the included literature. As a result, we identified 683 potentially relevant titles in our search and selected 51 studies in our review, reflecting “bio-think-tank” issues.

## Results

### Definition: What Is “Bio-Think-Tank”?

The “Bio-think-tank” distinguishes itself from existing policy-oriented or translational research by not merely informing policy through scientific findings, but by actively integrating policy translation mechanisms and governance frameworks into its core operation, functioning as a strategic knowledge hub for public health decision-making. Unlike traditional translational research which often follows a linear path from bench to bedside to policy, the bio-think-tank employs a dynamic, iterative process. It encompasses real-time data analytics, AI-driven predictive modeling, and institutionalized communication channels to policymakers, ensuring evidence-based recommendations are not just available, but directly integrated into both immediate crisis response and long-term health system planning. This active, embedded role within policy ecosystems, coupled with a focus on comprehensive governance and ethical frameworks, provides a sharper analytical scope that extends beyond conventional research translation.

At its core, the Bio-think-tank operates at the intersection of science, policy, and governance. It not only collects and curates longitudinal biological, genomic, and environmental data but also deploys advanced computational tools—such as artificial intelligence and predictive modeling—to generate timely, policy-relevant insights [35]. These insights are systematically communicated to policymakers, public health agencies, and international organizations through institutionalized channels, ensuring that evidence-based recommendations inform both immediate crisis response and long-term health system planning.

Moreover, a bio-think-tank embeds values of transparency, equity, and public trust into its operational model by aligning data governance with internationally recognized standards such as the FAIR principles—ensuring that data and biological materials are Findable, Accessible, Interoperable, and Reusable. Originally articulated to enhance the utility and reproducibility of scientific data, the FAIR framework has increasingly been applied to biobanking as a normative foundation for responsible data stewardship and equitable access [36]. By advancing open yet governed access to high-quality data, a bio-think-tank promotes fair-use data governance while systematically incorporating ethical, legal, and societal considerations into knowledge production [37]. Finally, as a node within global health governance, such an institution facilitates international collaboration through methodological interoperability and federated data-sharing models, while committing to equitable benefit-sharing arrangements that mitigate risks of data extractivism and reinforce global research solidarity.

The “Bio-think-tank” represents a paradigm shift from static biobanking toward dynamic, policy-engaged infrastructures, positioning itself as both a scientific engine and a strategic partner in shaping national and global public health policy. Based on the interpretation of functionalist theory and the analysis of existing literature, Table 1 provides a comparative summary of the distinctions between “Biobank” and “Bio-think-tank” across nine dimensions: core function, data orientation, policy integration, governance, public trust, global role, crisis responsiveness, and strategic vision.

**TABLE 1:** Comparison between “biobank” and “bio-think-tank” (Switzerland, 2025).

Dimension	Biobank (current model)	Bio-think-tank (future model)
Core function	Biomedical data repository for hypothesis-driven research	Adaptive knowledge hub for real-time policy guidance
Data orientation	Longitudinal, large-scale, but demographically skewed	Representative, interoperable, with AI-assisted quality control
Policy integration	Indirect, via academic publications and slow uptake	Direct, with policy liaison units, briefs, and decision-ready tools
Governance	Research-focused, centralized access with limited transparency	Multi-stakeholder, participatory, and accountable governance
Intermediate/Hybrid models (e.g., research consortia, data federations)	Partially integrated, project-specific data sharing, limited policy mandate	Integrates elements of both, with a clear mandate for policy translation and active engagement in health governance, often operating through federated data networks
Public trust	Conditional, hindered by concerns of bias and data use opacity	Reinforced through transparency, feedback loops, and citizen engagement
Global role	National asset with international research collaborations	Strategic leader in federated biobank networks and global health governance
Crisis responsiveness	Valuable but reactive (e.g., COVID-19 insights)	Proactive real-time surveillance, predictive modeling, and scenario testing
Strategic vision	Scientific excellence and productivity	Integrated policy partner shaping national and global health security

### Why Does the UK Biobank Need to Achieve Functional Evolution?

The COVID-19 pandemic has revealed both the strengths and the limitations of existing biomedical data infrastructures such as the UK Biobank. While the UK Biobank’s vast repository of biological samples, genomic data, and lifestyle information proved valuable in identifying COVID-19 risk factors and exploring long-term sequelae [38, 39], the crisis also demonstrated that passive data repositories cannot fully meet the real-time policy needs of a rapidly changing public health landscape.

Firstly, in the post-pandemic era, the urgency of integrating epidemiological surveillance, predictive modeling, and rapid policy translation has grown. Scholars note that a “static” biobank model limits responsiveness to emerging threats, as it was designed primarily for long-term, hypothesis-driven research rather than rapid scenario testing and decision support [40]. Upgrading to a “bio-think-tank” would mean embedding real-time analytics, AI-based risk stratification, and direct communication pipelines to public health agencies, thus turning the UK Biobank into a proactive rather than reactive player in health governance. The shift aligns with the WHO’s call for more adaptive, knowledge-driven infrastructures capable of cross-sectoral coordination in future health emergencies [41].

A second rationale for transformation lies in the growing expectation that large-scale biomedical infrastructures should serve not only the scientific community but also policymakers, especially in contexts where health inequalities and population vulnerabilities are amplified by crises. The UK Biobank’s current structure provides data access mainly to qualified researchers, but lacks systematic mechanisms to translate findings into actionable guidance for legislators and health administrators. By evolving into a bio-think-tank, the UK Biobank could establish dedicated policy liaison units, produce real-time policy briefs, and generate risk assessments tailored to regional and demographic contexts. Such a role is particularly important in the post-pandemic setting, where governments are balancing recovery planning, economic stability, and preparedness for future outbreaks [42]. Furthermore, scholars have highlighted that integrating legal, ethical, and societal perspectives into data-driven public health recommendations increases both legitimacy and public trust [43].

Finally, a strategic imperative lies in consolidating the UK Biobank’s position as a global frontrunner within an increasingly competitive biobanking landscape. Over past decades, leading initiatives in the US, the EU, China, and beyond have expanded their infrastructures by integrating AI-driven analytics, federated data-sharing architectures, and robust public engagement frameworks. Against this backdrop, the UK Biobank must not only sustain its comparative advantages but also adapt to evolving international benchmarks that increasingly define excellence in biobank governance, technological sophistication, and societal legitimacy. Scholars have emphasized that post-pandemic health diplomacy will increasingly revolve around data governance, equitable access to predictive health tools, and the ability to model transnational risks [44]. A Bio-think-tank model would allow the UK Biobank to act not only as a data provider but as a thought leader shaping international norms for biobank governance, bias mitigation, and algorithmic transparency. This could also support the creation of a global biobank network capable of harmonizing methodologies and accelerating collaborative research, ensuring that lessons from COVID-19 translate into long-term resilience against future pandemics. Thus, the transformation is not just a national upgrade but a strategic repositioning in the international health policy arena.

## Discussion

The analysis suggests that the functional remit of the UK Biobank ought to expand beyond its traditional role as a research infrastructure to encompass a more active contribution to public health policymaking and legislative design. Recasting the biobank as a “bio-think-tank” resonates with the UK government’s broader strategic objectives in strengthening both national and global health security. The US All of Us Research Program exemplifies the political and scientific value of integrating diverse population datasets into public health planning, thereby establishing a benchmark for globally competitive biobank governance [45]. Within Europe, the BBMRI-ERIC network has fostered a federated infrastructure that facilitates cross-border research and consolidates the role of biobanks in shaping European health policy [46]. Meanwhile, China has pursued a strategy of large-scale investment in genomic and health data infrastructures, with the China Kadoorie Biobank and a growing constellation of provincial initiatives rapidly expanding national capacity and signaling its ambition to become a global leader in precision health research [47]. Against this international backdrop, the UK Biobank confronts a dual imperative: maintaining its reputation for scientific excellence while evolving into a strategic resource for health policy. Its future development will hinge on several critical priorities.

### Optimization of Sample and Data Quality

It is essential that public health policies be grounded, wherever feasible, in evidence generated through scientifically rigorous processes—that is, data collected and analyzed in a systematic, methodologically robust manner [48]. High-quality biological samples and robust datasets are the foundation of a credible biobank. The UK Biobank already maintains rigorous collection, processing, and storage protocols, ensuring that its biological materials meet research-grade standards [49]. However, transitioning to a bio-think-tank demands an even higher level of precision—where datasets are representative, longitudinally consistent, and interoperable with other major biobank systems [50].

One major challenge is participant diversity. Current data reveal a demographic skew towards middle-aged, white, and healthier-than-average individuals. This imbalance limits the generalizability of findings for ethnic minority groups and for conditions that disproportionately affect disadvantaged populations. In the context of AI-driven health analytics, this bias can have amplified downstream effects—such as reinforcing inequities in health risk prediction models [12]. Therefore, the UK Biobank should adopt targeted recruitment strategies to include underrepresented communities.

Interoperability constitutes a further critical dimension. Aligning metadata structures and variable definitions with internationally recognized standards—such as those advanced by the Global Alliance for Genomics and Health — is essential to facilitating integration into multinational research consortia [51]. This is not merely a technical exercise; rather, the comparability of datasets across jurisdictions is a precondition for effective international policy collaboration and for the realization of truly global public health governance.

Moreover, to ensure that policy decisions based on biobank data are sound, quality control systems must include real-time anomaly detection, missing-data alerts, and AI-assisted data validation pipelines [52]. Even small errors—such as misclassified lifestyle factors—can distort epidemiological models and lead to suboptimal policy interventions. By strategically expanding participant diversity, adopting interoperable standards, and investing in advanced quality assurance, the UK Biobank can lay the groundwork for evidence that is both scientifically rigorous and policy-ready.

### Build a Bridge Between Scientific Judgment and Public Trust

From a functionalist perspective, the transformation of UK Biobank should enhance its functional assumption among the general public, fostering expectations for the joint achievements of policymakers and researchers. Public trust is a prerequisite for the legitimacy of any biobank seeking a policy advisory role [53]. Striking the right balance between values of personal and familial privacy, dignity, personal choice and non-discrimination while maintaining transparency and explainability is essential for fostering public trust, involvement and participation in AI-driven public health biobanks [54]. Without trust, participation rates decline, data sharing slows, and policy uptake of biobank findings is hindered [55]. In the UK, trust hinges on transparency in governance, clarity about data uses, and genuine opportunities for public involvement [56]. A lack of transparency can severely damage public trust in data-intensive projects like research biobanks, as shown by the unsuccessful attempt to roll out the Care.Data program in NHS England [57]. Public health organizations play a crucial role in developing data governance frameworks that guarantee accountability and transparency, all while harnessing the power of data and AI for the benefit of society [58]. Experts further emphasize the necessity of reinforcing accountability mechanisms within biobanks, advocating for their implementation in an open, accessible, and proactive manner to facilitate stakeholders' and the public’s comprehension of biobank operational frameworks [59].

Simply adhering to the law is not enough to build public trust; continuous and thorough public consultation aimed at jointly developing standards is essential, especially when dealing with emerging technologies whose social impacts are still uncertain [60]. Building this bridge requires structured engagement infrastructures—such as citizen assemblies and deliberative panels—that allow for reciprocal dialogue between scientists and the public [61]. Feedback loops are equally important. When participants see that their data has contributed to a change in health policy or improved screening programs, trust deepens and willingness to engage increases [62].

Finally, scientific judgment must be communicated in a way that is both accurate and accessible [63]. Overly technical reports risk alienating lay audiences, while oversimplification risks distortion. Developing communication strategies that use plain language, visual aids, and contextual explanations can make complex genomic and epidemiological insights understandable without sacrificing nuance [64].

### Enhance the Accessibility and Utilization of Data for Policymakers

To influence health policy effectively, the UK Biobank must ensure its data are not only accessible to scientists but also usable by policymakers under time and resource constraints. Policymakers often require synthesized insights rather than raw data, with clear explanations of uncertainty and relevance to specific policy contexts [65]. Creating policy-facing analytical tools—such as interactive dashboards, real-time epidemiological trackers, and AI-powered scenario simulations—can bridge the gap between raw data and decision-ready intelligence. The application of advanced information technology provides continuous motivation for the sustainable development of biobanks, improves data quality by enhancing data collection processes, streamlines data management systems, and facilitates more sophisticated analytical techniques. In addition, it mitigates human error and increases the efficiency of data management using artificial intelligence algorithms [66].

Moreover, embedding data scientists directly within government health departments can also promote a shared understanding of capabilities and limitations. This structural integration reduces the risk of misinterpreting findings—particularly in politically sensitive areas like resource allocation or health inequality [12]. By rethinking data dissemination for policy contexts, the UK Biobank can extend its impact from informing academic publications to shaping real-world health strategies. However, the Bio-think-tank model might not be universally applicable and is particularly suited for large, population-based biobanks like the UK Biobank, while its application to other types would require careful adaptation.

### Strengthen Partnerships Among Public Health Experts, Researchers, Data Scientists and Stakeholders From Various Sectors

The transition to a bio-think-tank cannot be achieved in isolation. Intersectoral networks and coordination are gaining traction, with claims that they represent ways to reorganize and transform public services within a new, collaborative framework of governance [67]. Multi-sector partnerships create a richer and more agile knowledge ecosystem, blending epidemiological insight, methodological innovation, and societal relevance [68].

Within the UK, this means connecting public health agencies, NHS trust, universities, technology companies, and patient advocacy groups in structured collaborations. Internationally, networks like Biobanking and Biomolecular Resources Research Infrastructure – European Research Infrastructure Consortium (BBMRI-ERIC) demonstrate that shared governance and pooled resources can accelerate discovery while ensuring equitable access to outputs [46].

Cross-sectoral partnerships also bring in new capabilities. Data scientists from the private sector can introduce novel machine learning methods, while public health experts ensure that outputs align with population health priorities [69]. By embedding partnership-building into its strategic mission, the UK Biobank can position itself as both a national resource and an international convenor for collaborative, policy-relevant research.

### Disadvantages of Bio-Think-Tank

Despite its strategic appeal, repositioning the UK Biobank as a “bio-think-tank” entails several substantive disadvantages that warrant careful consideration. Most notably, there is a significant risk of mission creep: expanding from a neutral, long-term research infrastructure into an active policy-oriented actor may blur the boundary between evidence generation and policy advocacy. Such role expansion could divert institutional attention and resources away from core functions—such as data stewardship, methodological rigor, and open-ended scientific inquiry—toward short-term policy responsiveness, thereby weakening the UK Biobank’s scientific credibility. Closely related is the heightened exposure to political pressures that accompanies deeper engagement with policymaking. As the UK Biobank becomes more visible in politically sensitive debates over health inequalities, resource allocation, or emerging technologies, its findings may be selectively interpreted or instrumentalized by political actors, potentially undermining perceptions of neutrality and public trust. Moreover, fluctuating political priorities risk destabilizing the continuity required for longitudinal research infrastructures. Finally, the operational complexity of integrating diverse forms of expertise presents a nontrivial challenge. A bio-think-tank model necessitates sustained collaboration among scientists, data analysts, ethicists, legal experts, and policymakers, each operating with distinct epistemic frameworks and professional incentives. Without robust coordination mechanisms, such interdisciplinarity may generate inefficiencies, internal tensions, or the dominance of technical perspectives at the expense of ethical and societal considerations. Collectively, these risks underscore the need for clearly defined institutional boundaries, strong governance safeguards, and sustained investment in interdisciplinary capacity if the bio-think-tank model is to enhance—rather than compromise—the UK Biobank’s long-term value.

### A Model for the Global Biobank as a Vision

The culminating stage in the evolution of the UK Biobank is its articulation as a benchmark for the global biobanking community. Achieving this requires not only compliance with international best practices in ethics, governance, and technical interoperability, but also active leadership in shaping them. Existing frameworks—such as the Organization for Economic Co-operation and Development (OECD) Guidelines on Human Biobanks and the Global Alliance for Genomics and Health’s (GA4GH) data-sharing principles—provide a foundational reference point. Yet, to ensure that governance structures keep pace with the accelerating globalization of biotechnologies, governments must collaborate to establish new institutional mechanisms and regulatory frameworks capable of sustaining legitimacy, accountability, and cross-border trust. The World Health Organization has called on governments around the world to work together to establish new institutional structures and rules to ensure that international governance keeps pace with the globalization of technology [70]. A global leadership role would entail active participation in federated data networks—systems where datasets remain locally controlled but can be queried internationally under standardized governance rules. This approach respects jurisdictional differences in privacy law while enabling cross-border research on global health challenges.

Equity is central to this vision. Global data sharing must avoid reproducing the exploitative patterns of “data colonialism,” where low- and middle-income countries contribute data without gaining proportional benefits [71]. The UK Biobank could set a precedent by committing to capacity-building partnerships, technology transfers, and co-authorship policies that ensure fair recognition of all contributors. If successfully implemented, the UK Biobank could become not just a national asset but a diplomatic tool—advancing health science while also strengthening the UK’s role in global health governance.

### Conclusion

The evolution of the UK Biobank from a large-scale biomedical repository toward a proactive “bio-think-tank” epitomizes the shifting demands of contemporary public health governance and the expanding role of scientific infrastructures in the 21st century. Originally established to elucidate the complex interplay between genetic and environmental determinants of health, the UK Biobank has generated unparalleled longitudinal and multidimensional datasets that have supported significant advances in disease prevention, the analysis of health inequalities, and the technological modernization of public health policy. Nonetheless, the findings of this study underscore critical limitations in its governance framework, translational pathways, and population representativeness, which collectively constrain its capacity to function as a direct and authoritative driver of evidence-based policy.

From a functionalist perspective, the UK Biobank’s functional cognition is firmly established within both scientific and policy communities: it is regarded not simply as a genomic repository, but as a comprehensive and integrated health data infrastructure. Its functional assumption—that it can directly underpin targeted, equitable, and timely public health interventions—remains only partially realized. In terms of functional performance, the UK Biobank has achieved notable success in academic productivity and scientific discovery, yet its translation into policy impact is uneven.

The future relevance of the UK Biobank hinges on its capacity to integrate epidemiological surveillance, AI-enabled predictive analytics, and agile communication pipelines into its operational framework. Such integration would enable a transformation from a predominantly hypothesis-driven repository into a dynamic policy partner, capable of supporting rapid responses to acute public health crises as well as addressing long-term structural challenges. The experience of COVID-19 underscores the value of this adaptive model. More broadly, the vision of the UK Biobank as a “bio-think-tank” reflects a wider transition toward knowledge-driven health governance, in which scientific infrastructures function not merely as passive data providers but as active contributors to policy design and public trust-building. If pursued with methodological rigor, inclusivity, and international cooperation, this trajectory would position the UK Biobank as both a national cornerstone of evidence-based policymaking and a global leader in health data governance—setting an international benchmark for how biobanks can advance equitable, scientifically robust, and globally interconnected public health policy.

### Outlook and Limitations

Building on this analysis, the article outlines several concrete and incremental actions through which the UK Biobank could operationalize its transition toward a Bio-think-tank role. These include piloting a dedicated policy liaison and translation unit to institutionalize engagement with government departments and public health agencies; developing rapid-response analytical and briefing mechanisms around priority health risks to better align scientific outputs with policy timelines; and initiating structured public engagement activities, such as citizen panels, participatory consultations, and transparent communication platforms, to strengthen social legitimacy and trust in data governance. Furthermore, sufficient and sustainable financial support constitutes a critical element for the establishment and operation of Bio-think-tank. Exploring diversified funding sources may serve as an effective solution, such as public-private partnerships and levying fees on commercial data users. Taken together, these steps are intended not to displace the biobank’s core scientific mission, but to extend its functional reach within the public health decision-making ecosystem.

At the same time, several limitations of this study must be acknowledged. The analysis relies predominantly on English-language academic literature, policy documents, and publicly available materials, which may underrepresent perspectives from non-English sources or informal policy debates. In addition, some of the forward-looking recommendations advanced here are necessarily speculative and normative in nature, particularly with respect to institutional reform and governance innovation. Their practical feasibility remains contingent on political priorities, resource availability, and organizational culture, and thus cannot be fully substantiated within the scope of the present study.

In light of these constraints, future research should adopt more empirical and stakeholder-oriented approaches. Subsequent work will seek to conduct interviews or surveys with key UK Biobank stakeholders, including senior administrators, affiliated researchers, policymakers, and public representatives, to collect first-hand evidence on the feasibility, desirability, and perceived risks of the Bio-think-tank model. Such data would enable a more grounded assessment of how scientific infrastructures can reposition themselves within contemporary health governance, and would provide critical insight into the conditions under which evidence generation, policy translation, and public accountability can be more effectively aligned.
